# Clinico-molecular predictors of durable response to immune checkpoint inhibitors (ICI) in metastatic cervical cancer (mCC)

**DOI:** 10.1038/s41416-026-03438-6

**Published:** 2026-05-19

**Authors:** S. Barraud, C. Roussel-Simonin, P. Pautier, A. Italiano, C. Massard, A. Hollebecque, J. Michels, F. Blanc-Durand, K. Ouali, E. Rouleau, A. Leary

**Affiliations:** 1 Department of Medical Oncology, Gustave Roussy, Villejuif, France; 2 Drug Development Department (DITEP), Gustave Roussy, Villejuif, France; 3 Department of Medical Biology and Pathology, Cancer Genetics Laboratory, Gustave Roussy, Villejuif, France

**Keywords:** Cancer genomics, Cancer

## Abstract

**Background:**

Immune checkpoint inhibitors (ICI) are approved for metastatic cervical cancer (mCC) after platinum failure, but only a minority of patients derive durable benefit. We aimed to compare long-term responders (LTR) and non–long-term responders (NLTR) to ICI and to identify clinical and molecular predictors of long-term response.

**Methods:**

We retrospectively reviewed patients with mCC treated with ICI at Gustave Roussy. LTR were defined as patients achieving complete or partial response or stable disease lasting >12 months, while NLTR progressed within 12 months. Logistic regression analyses were performed to identify factors associated with LTR. Genomic analyses were available for a subset of patients using large-panel next-generation sequencing.

**Results:**

Between January 2015 and September 2023, 106 patients received ICI; 20 (19%) were classified as LTR and 86 (81%) as NLTR. After a median follow-up of 42 months, median progression-free survival was not reached in LTR versus 3.5 months in NLTR. Eleven LTR maintained a sustained response after ICI discontinuation. In multivariate analysis, oligometastatic disease, lymph node–only metastases, and progression outside prior radiation fields were independently associated with LTR. Tumour mutational burden and mismatch repair status were not predictive. PIK3CA mutations were significantly more frequent in LTR, while STK11 alterations were enriched in NLTR.

**Conclusion:**

Durable responses to ICI in mCC, although uncommon, are associated with prolonged disease control and may persist after treatment discontinuation. Specific clinical and molecular features are strongly associated with long-term benefit and may help refine patient selection for immunotherapy.

## Introduction

Cervical cancer (CC) is the most prevalent gynaecological cancer worldwide, accounting for 604,127 new cases in 2020. It is also the fourth most common cancer among women. Despite substantial efforts at primary prevention, exemplified by HPV vaccination and secondary prevention through screening programmes, mortality rates remain unacceptably high [1], with 342,000 reported deaths and an age-standardised rate of 13.3 per 100,000 in 2020. In Europe, cervical cancer (CC) remains a significant cause of mortality among middle-aged women [2]. Five-year survival rates vary considerably, from 91% for localized disease to 19% for cases with metastatic spread [3]. As a malignancy associated with a failure to clear an HPV infection and immunosuppressed states, CC was expected to be a good candidate to immunotherapies. Despite a strong biological rationale, the activity of single agent anti-PD-1/PD-L1 inhibitors in patients with recurrent or metastatic cervical cancer (mCC) progressing post-platinum remains modest. Only 15% of patients demonstrate objective responses with a median progression-free survival of 3-4 months [4, 5]. In the frontline setting, the combination of pembrolizumab or atezolizumab with platinum-based chemotherapy improved median progression-free survival (PFS) and overall survival, but outcomes remain dismal with median PFS of 10 to 12 months [6, 7].

Despite the relatively modest benefits from ICI monotherapy, a subset of patients exhibits durable clinical benefit. Indeed, median duration of response (DOR) was 16.4 months with cemiplimab [4], and 18 months with pembrolizumab [8] compared to standard chemotherapy, although median follow-up was limited to 16.8 and 22 months, respectively. While tumour PD-L1 immunohistochemistry (IHC) and tumour mutational burden (TMB) have been extensively investigated in various histological cancer types, their applicability to CC remains unclear [9–13]. Cemiplimab improved overall survival regardless of PD-L1 expression, and pembrolizumab in combination with frontline chemotherapy displayed favourable outcomes in the intention-to-treat (ITT) population, thereby undermining the utility of PD-L1 IHC as a robust biomarker. The clinical relevance of TMB in CC has not yet been clearly established. Clinical and molecular features predictive of prolonged response, such as high TMB ( ≥ 50th percentile), PD-L1 ≥ 50% or non-epidermoid histological type have been reported in other solid tumors [14, 15].

We therefore conducted a retrospective study to describe long-term outcomes in patients treated with ICI and to identify clinical and molecular features associated with exceptional response to ICI in mCC.

## Material and methods

### Patients

All consecutive patients presenting with recurrent or de-novo metastatic CC treated with immunotherapy either as monotherapy or in combination with chemotherapy and/or an anti-angiogenic at Gustave Roussy between January 2015 and September 2023 were included.

Clinical data were retrospectively collected from the medical records, including age, Eastern Cooperative Oncology Group’s (ECOG) performance status (PS), smoking status, body mass index (BMI), histological subtype, biological markers (LDH, lymphocytes, neutrophils) and prior treatments received. High NLR was defined as ≥5, based on prior meta-analyses in non-small cell lung cancer demonstrating its prognostic significance for progression-free and overall survival [16, 17]. Stage at diagnosis was evaluated according to the 2018 International Federation of Gynecology and Obstetrics’s (FIGO) classification.

Patients were defined as: (1) Long-Term Responders (LTR), if they achieved complete response (CR), partial response (PR), or stable disease (SD) under ICI for a minimum of 12 months; and (2) Non-Long-Term Responders (NLTR), if they progressed within 12 months after ICI initiation. Oligometastatic disease was defined as the presence of ≤2 metastatic sites. For nodal involvement, pelvic lymph nodes were considered as a single metastatic site.

### Molecular analyses

Molecular data from circulating tumor DNA (ctDNA) and tissue analyses performed at Gustave Roussy within specific trials, STARTRK (NCT02097810), STING (NCT04932525), MOSCATO (NCT01566019) and RAGNAR (NCT04083976) were collected.

Each of these studies organized large-panel Next Generation Sequencing (NGS). In the STING trial, somatic alterations were identified by hybrid capture-based targeted DNA sequencing using the FoundationOne Liquid CDx Assay (324 genes, tumour mutational burden, microsatellite instability status). In the STARTRK and RAGNAR trials, somatic alterations were identified by hybrid capture-based targeted DNA sequencing using the FoundationOne® CDx Assay (321 genes, tumour mutational burden, microsatellite instability status). In the MOSCATO trial, somatic alterations were identified by targeted sequencing and array comparative genomic hybridization (aCGH) analysis. RNA-sequencing analyses were also performed.

### Ethical considerations

This study was conducted in accordance with the ethical principles of the Declaration of Helsinki of 1964 and its subsequent revisions and with Good Clinical Practice of the International Council for Harmonization (ICH–E6, 17/07/96). The protocol received approval from the Scientific Commission of Gustave Roussy (French ethic committee) on 13 March 2023. An information letter was sent to all patients included in the study who were still alive at the time of mailing.

### Statistical analysis

Continuous variables were summarised as mean ± standard error (SE) or median, while categorical variables were described using frequencies and percentages. Comparisons of continuous variables were performed using the Wilcoxon rank-sum test, and comparisons of categorical variables were performed using the chi-square test or Fisher’s exact test, as appropriate.

Median follow-up was calculated using the reverse Kaplan–Meier method. Overall survival (OS) was defined as the time from the first ICI infusion to death from any cause. Progression-free survival (PFS) was defined as the time from the first ICI infusion to first documented disease progression (RECIST or clinical progression) or death from any cause, whichever occurred first. Patients alive without disease progression were censored at the date of last follow-up. Median and 5-year OS and PFS were reported with 95% confidence intervals (CI). Disease control rate (DCR) was defined as the proportion of patients achieving CR, PR, or SD and was assessed according to RECIST version 1.1. Survival curves were estimated using the Kaplan–Meier method, and hazard ratios were estimated using Cox proportional hazards models.

Associations between response status and potential prognostic factors were assessed using logistic regression models. Univariate analyses were first performed, followed by multivariate logistic regression analyses adjusted for clinically relevant variables defined a priori, corresponding to the baseline characteristics reported in Table 1. These variables included age, histological subtype, FIGO stage, body mass index, ECOG performance status prior to immune checkpoint inhibitor initiation, metastatic sites, presence of oligometastatic disease, relapse within prior radiation fields, number of prior treatment lines, receipt of immunotherapy combined with chemotherapy, and the sum of target lesion diameters. Associations between OS and PFS and potential prognostic factors were evaluated using the log-rank test for univariate analyses and Cox proportional hazards regression models for multivariate analyses. All statistical tests were two-sided, and a *p* value < 0.05 was considered statistically significant.

**Table 1:** Characteristics of the patients at baseline, for all participants and by response.

Variable	Whole cohort	LTR	NLTR	*p*-value^a^
	*N* = 106	*N* = 20	*N* = 86	
**Age (years)**	49.85 (12.05)	55.86 (12.11)	48.45 (11.66)	0.020
**Histological subtype**				0.24
Adenocarcinoma	22 (20.8%)	2 (10.0%)	20 (23.3%)	
Squamous-cell carcinoma (SCC)	84 (79.2%)	18 (90.0%)	66 (76.7%)	
**FIGO**				0.23
I	17 (17.0%)	3 (15.8%)	14 (17.3%)	
II	20 (20.0%)	1 (5.3%)	19 (23.5%)	
III	36 (36.0%)	10 (52.6%)	26 (32.1%)	
IV	27 (27.0%)	5 (26.3%)	22 (27.2%)	
Unknown	6	1	5	
**BMI**				0.48
<18	11 (11.3%)	1 (5.6%)	10 (12.7%)	
>25	34 (35.1%)	5 (27.8%)	29 (36.7%)	
18–25	52 (53.6%)	12 (66.7%)	40 (50.6%)	
Unknown	9	2	7	
**PS (ECOG) prior ICI**				0.51
0	56 (54.9%)	16 (80%)	40 (46,5%)	
1	42 (41.2%)	0	42 (48,8%)	
2	4 (3.9%)	4 (20%)	0	
Unknown	4	0	4 (4,7%)	
**Metastatic sites**				
Lung	39 (36.8%)	6 (30.0%)	33 (38.4%)	0.48
Bone	15 (14.2%)	3 (15.0%)	12 (14.0%)	>0.99
Brain	1 (0.9%)	0 (0.0%)	1 (1.2%)	> 0.99
Liver	15 (14%)	1 (7%)	14 (93%)	0.45
Lymph nodes	75 (70.8%)	16 (80.0%)	59 (68.6%)	0.31
Lymph nodes only	11 (10.4%)	7 (35.0%)	4 (4.7%)	<0.001
**Oligo-metastatic disease**	12 (11.3%)	7 (35.0%)	5 (5.8%)	0.001
**Relapse in radiation fields**	43 (45.3%)	5 (27.8%)	38 (49.4%)	0.1
**Neutrophil to lymphocyte ratio** > **5**	85 (82.5%)	18 (90.0%)	67 (80.7%)	0.51
**LDH above normal limite**	6 (6.0%)	0 (0.0%)	6 (7.4%)	0.59
**Number of prior lines before immunotherapy**				0.4
0	18 (17.0%)	5 (25.0%)	13 (15.1%)	
1	46 (43.4%)	6 (30.0%)	40 (46.5%)	
>= 2	42 (61.3%)	9 (45.0%)	33 (38,4%)	
**Immunotherapy received with chemotherapy**	8 (7.5%)	2 (10.0%)	6 (7.0%)	0.64
**Sum of target lesions (size)**	55.29 (36.44)	38.21 (24.74)	59.68 (37.77)	0.008

^a^Wilcoxon rank sum test; Fisher’s exact test

All statistical analyses were performed using R software (version 4.3.1). Volcano plots were generated using the R package *ggplot2*.

## Results

### Patients’ characteristics

**Fig. 1:**
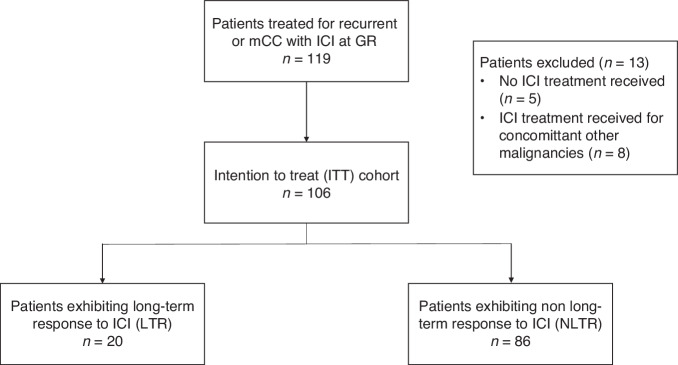
Flow chart of study population and ICI response groups. Flow chart.

A total of 106 patients were included (Fig. 1). Most of these patients (93, *n* = 88%) were included in clinical trials, mainly phase-1 studies. The vast majority received a chemotherapy-free regimen (93%) and had received prior chemotherapy for metastatic disease (83%). 8 patients received immunotherapy in combination with chemotherapy. Six patients received PD-L1 inhibitor in combination with CTLA-4 inhibitors. Fourteenpatients received PD-L1 inhibitor in combination with a VEGF inhibitor. Thirty-two patients received a PD-L1 inhibitor in combination with another ICI (such as Inducible Co-Stimulator (ICOS) or T cell immunoreceptor with Ig and ITIM domains (TIGIT) inhibitors). Finally, most patients received a single agent PD-1 or PD-L1 inhibitor (47 patients).

Taking the whole group, response rates (RR), PFS and OS were consistent with that observed in clinical trials. After a median follow-up of 36 months, median PFS was 3.9 months (Supplementary Fig. 1), median OS was 11.2 months (Supplementary Fig. 2) and DCR was 57.3% (Supplementary Table 1).

### Clinical outcomes for LTR vs NLTR

Twenty patients (19%) were classified as LTR and 86 as NLTR (81%). Among LTR, 35% achieved durable CR, 50% achieved PR for >12 months, and 15% achieved SD > 12 months. Among NLTR, 13.3% had short-term PR (Supplementary Table 1).

**Fig. 2:**
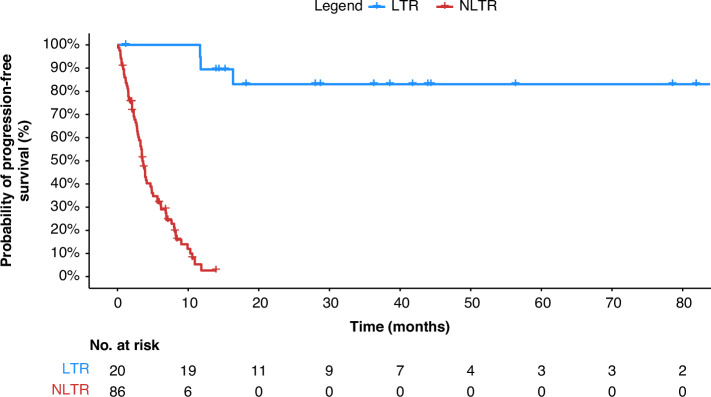
Kaplan-Meier curves for progression-free survival (PFS). Defined according to the Response Evaluation Criteria in Solid Tumors, version 1.1. Tick marks indicate censored observations, and vertical lines indicate the times of landmark PFS analyses. LTR Long term responders, NLTR Non long term responders.

After a median follow-up of 42 months in the LTR cohort [95% CI 28 - 78.6], median PFS was not achieved. In comparison, in the NLTR cohort, after a median follow-up of 26 months [95% IC 22.1 - NA], median PFS was 3.5 months, *p* < 0.001 (Fig. 2). LTR status was significantly associated with PFS compared to NLTR. Supplementary Figure 3 shows the density plot for PFS in the whole cohort.

Median overall survival was not reached in the LTR cohort after a median follow-up of 41.8 months versus 8.8 months in the NLTR cohort after a median follow-up of 26 months, *p* < 0.001 (Supplementary Fig. 4). LTR status was significantly associated with overall survival compared to NLTR: HR = 0.07 [95% CI 0.02 - 0.22], *p* < 0.001. Supplementary Figure 5 shows the density plot for OS in the ITT cohort.

Finally, among the 20 LTR, 11 patients were in sustained response with a median of 22 months (range 2-55mo) since ICI discontinuation.

### Clinical features associated with LTR

Baseline characteristics of the whole cohort (*n* = 106), the LTR cohort (*n* = 20), and the NLTR cohort (*n* = 86) are summarised in Table 1. The overall median age was 50 years, with LTR patients significantly older than NLTR (median 55 vs 49 years, *p* = 0.02). The most common histological subtype was squamous cell carcinoma (SCC) in both groups.

The proportion of patients with de novo metastatic disease (FIGO stage IV) was similar between LTR and NLTR at 27%. No significant differences were observed for ECOG-PS before ICI, baseline BMI, LDH, neutrophil-to-lymphocyte ratio, or number of prior lines (Table 1). The proportion of patients with relapse in prior radiation fields was not significantly different between LTR and NLTR (28% vs 49%, *p* = 0.1).

LTR demonstrated a significantly higher proportion of exclusively nodal metastases (7 patients, 35%) compared with NLTR (4 patients, 5%; *p* < 0.001). Oligometastatic disease was more common in LTR (*n* = 7, 35%) than NLTR (*n* = 5, 6%; *p* = 0.001). In contrast, liver metastases were more frequent in NLTR (14 patients, 13%) compared with LTR (1 patient; *p* < 0.001).

**Fig. 3:**
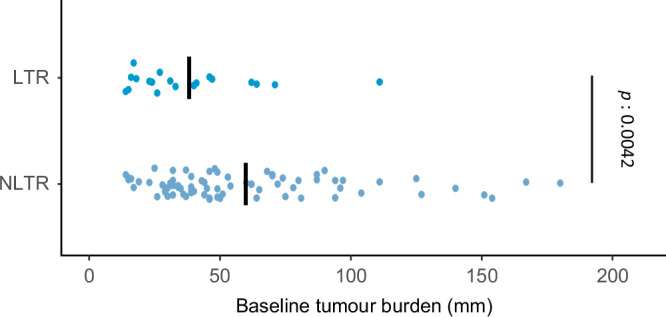
Comparison of baseline tumour burden between LTR and NLTR groups Baseline mutational burden, by response (Wilcoxon test).

LTR patients also had significantly lower tumour burden (median sum of target lesions 38 mm vs 60 mm in NLTR, *p* = 0.004, Fig. 3). The proportion of patients receiving ICI with chemotherapy was negligible in both groups ( < 10%).

### Predictors of long-term response

Logistic regression identified three parameters significantly associated with LTR: lymph node-only metastasis (HR = 8.23, 95% CI 1.36–56.2, *p* = 0.023), oligometastatic disease (HR = 11.4, 95% CI 2.15–72.4, *p* = 0.006), and absence of recurrence in radiation fields (HR for recurrence = 0.17, 95% CI 0.03–0.81, Table 2).

**Table 2:** Logistic regression of baseline characteristics associated with long-term responder (LTR) status

Characteristic	Multivariate	95% CI	*p*-value
	OR		
**Age**			
> 50 years old	3.16	0.86, 13.7	0.10
**Lung metastasis**			
Presence	0.41	0.08, 1.96	0.3
**Liver metastasis**			
Presence	0.43	0.02, 3.38	0.5
**Peritoneal metastasis**			
Presence	0.56	0.26, 1.22	0.14
**Lymph node metastasis only**			
Presence	8.20	1.36, 56.2	**0.023**
**Oligometastasis**			
≤ 2	11.4	2.15, 72.4	**0.006**
**Sum of target lesion (mm)**			
Above median	1.06	0.28, 4.17	>0.9
**Neutrophil to lymphocyte ratio (NLR)**			
> 5	1	1.38	0.20, 23.3
**Relapse in radiation fields**			
Yes	0.17	0.03, 0.81	**0.033**

*OR* Hazard Ratio, *CI* Confidence Interval

The values in bold correspond to those with a significance level of *p* 0.001.

**Fig. 4:**
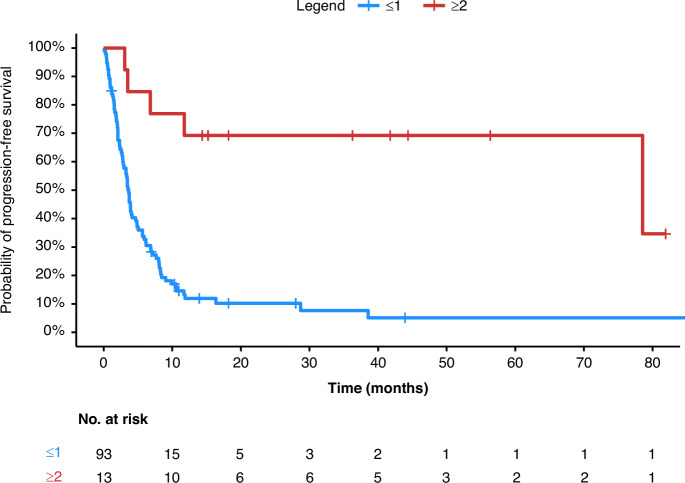
Kaplan–Meier curves for progression-free survival (PFS) according to score category. Three parameters are considered and each account for 1 point: absence of relapse in radiated fields, oligo-metastatic disease, and lymph node metastasis only.

A composite score incorporating these three factors was created, and PFS and OS were evaluated according to the presence of ≥2 criteria. Patients with ≥2 parameters (*n* = 13) had a median PFS of 79 months (95% CI 12–NA) versus 3.7 months (95% CI 3.1–4.9) in patients with <2 parameters (*n* = 93, *p* < 0.001, Log-Rank test, Fig. 4).

### Molecular analysis

**Fig. 5:**
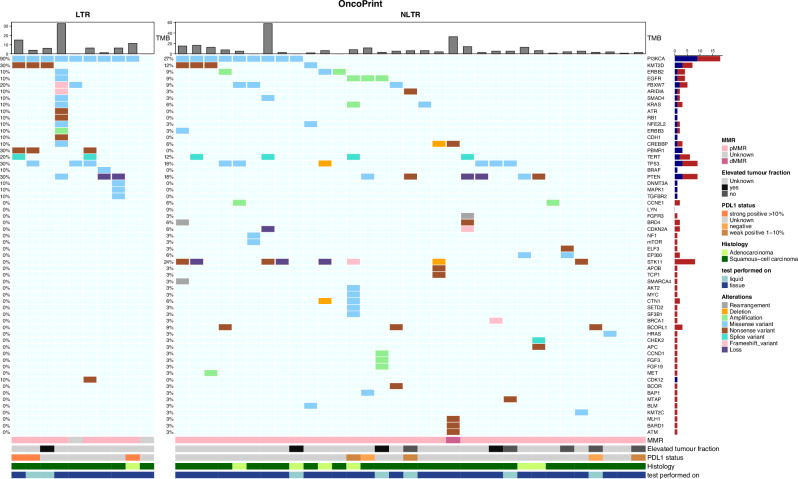
Oncoprint representing most expressed genes in the two cohorts. *NLTR* Non Long Term Reponders, *LTR* Long Term Responders

Molecular testing was conducted on a total of 43 patients, comprising 10 patients in the LTR group and 33 patients in the NLTR group. The majority of tumours harboured alterations in the PIK3CA–AKT–mTOR pathway. Notably, 40% had *PIK3CA* pathogenic variants (PVs), and 20% had *PTEN* loss due to mutation or deletion. Other frequent PVs included *KMT2D* (18%), *TP53* (18%), *STK11* (18%), and *TERT* (15%) (Fig. 5).

**Fig. 6:**
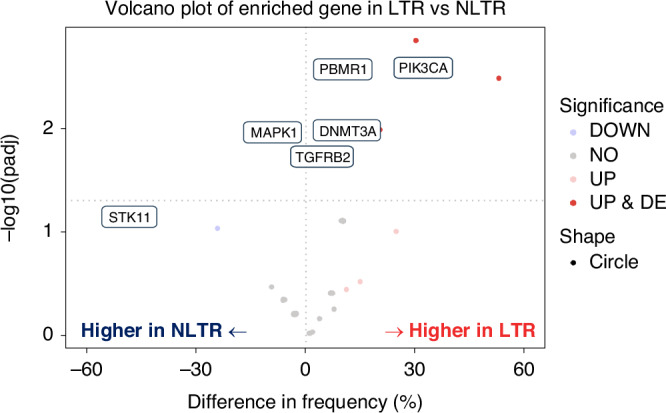
Volcano plot of enriched genes in long term responders (LTR) versus non-long term responders (NLTR). Plot depicts difference in frequency of bindividual gene alterations between LTR and NLTR (x-axis) versus -log10 (*P*) for Fisher exact test between LTR and NLTR groups (y-axis). Significance Categories: DOWN: Genes that are significantly less frequent in LTR (blue dots). NO: Genes that show no significant difference between LTR and NLTR (grey dots). UP: Genes that are more frequent in LTR but not differentially expressed (light red dots). UP & DE: Genes that are both more frequent in LTR and differentially expressed (red dots). Shape: All data points are represented as circles.

Comparing gene alteration frequencies between the two groups, LTR had significantly more *PIK3CA* mutations (78% vs 29%, *p* = 0.0014) and *PBRM1* mutations (22% vs 3%, *p* = 0.003) (Fig. 6). LTR also tended to present higher rates of *PTEN* loss (33% vs 16%) and *KMT2D* mutations (33% vs 13%), although these differences were not statistically significant. Similarly, several commonly altered and biologically relevant genes—*BRAF, ATR, CDH1, RB1, NFE2L2*, and *CREBBP*—showed trends toward higher prevalence in LTR without reaching significance. In contrast, NLTR patients showed enrichment of PVs in *STK11* (24% vs 0%).

No significant differences were observed in tumour mutational burden (TMB) or mismatch repair (MMR) status between LTR and NLTR patients.

## Discussion

To our knowledge, this is the first cohort to describe these long-term responders to ICI in cervical cancer. We reported that 19% of metastatic cervical cancer patients treated with ICI demonstrated long-term response. These patients had significantly prolonged survival, as evidenced by an unreached median progression-free survival after a median follow-up of 42 months. Importantly, approximately half of these patients remained in sustained response years after ICI discontinuation. We then identified several clinical features overrepresented in these patients, like oligo-metastatic disease, the presence of exclusive nodal metastases and the absence of relapse in the radiation fields. Based on these parameters, we established a simple scoring system, which was able to predict responder status to immunotherapy and accurately identified 13 of the 19 LTR patients (68%). Molecularly long-term responders showed a higher prevalence of *PIK3CA* and *PBMR1* mutations compared to NTLR. The mutation rate in our global cohort was similar to published data [18]. Mutations in *PIK3CA* have been reported to be associated with an immune-related pathway that may predict response to ICI in breast cancer [19]. The second most upregulated gene was *PBMR1* in long-term responders (30%). Polybromo-1 (PBRM1) is part of the switch/sucrose nonfermenting (SWI/SNF) chromatin remodelling complex, and acts as a tumour suppressor gene in many cancers [20], and has been associated with response to ICI in many cancers [21, 22]. This gene and its tumour suppressor effect may be due to interactions with the tumour suppressors TP53 and/or EZH2 [23]. It has also been described as a potential biomarker for response to neoadjuvant chemotherapy in CC [24].

We used a 12-month cut-off to define patients with long-term response to immunotherapy. We chose this cut-off because the median progression-free survival with ICI used as monotherapy for recurrent or mCC is slightly less than 3 months. Several publications have addressed the issue of long-term responders to immunotherapy, mainly in lung cancer [14, 15]. The cut-off point chosen in these studies was 18 to 24 months to identify these patients, but survival rates in monotherapy trials in lung cancer are significantly higher than in cervical cancer [25–27].

We created a score based on clinical characteristics that may better select patients who may have a prolonged response to ICI. In cervical cancer, almost 50% of patients are diagnosed with locally advanced cervical cancer [28]. The standard of care at this stage is chemoradiotherapy [29], and relapse rates in the irradiated field are therefore common observed in 20-40% of the cases [30]. In this context, the absence of relapse in the irradiated fields was associated with LTR, as was the presence of nodal involvement only and oligo-metastases. Consequently, the presence of at least two of these three parameters was able to identify 13 of 19 of LTR patients.

Our study has certain limitations, mainly due to its retrospective nature. First, the total number of patients included (n = 106) and the low prevalence of LTR have limited the statiscal power of our study. In addition, we were not able to test our predictive score for PFS in a validation cohort.

Our observations warrant now an independent validation on a different cohort and ideally in the context of a prospective trial.

## Conclusion

Metastatic cervical cancer remains a major cause of mortality worldwide. Although responses to ICI in mCC remain rare ( < 20% patients), they are associated with impressive outcomes (median PFS > 3.5years) with a significant proportion disease-free months to years after ICI discontinuation. Our retrospective study unravelled several clinico-molecular features strongly associated with LTR and may help the identification of patients most likely to benefit from PD1/PDL1 inhibition alone or with chemotherapy, while others should probably: be oriented towards trials testing novel strategies. These data must now be confirmed, ideally prospectively, by a larger-scale study.

## Supplementary information

Supplementary Materials' legends

Supplementary Table 2. Somatic alterations

Supplementary Figure 1. Kaplan–Meier curves for progression-free survival (PFS), defined according to the Response Evaluation Criteria in Solid Tumors, version 1.1.

Supplementary Figure 2. Density Plot of Progression-Free Survival (PFS)

Supplementary Figure 3.

Supplementary Figure 4. Kaplan–Meier curves for overall survival (OS).

Supplementary Figure 5. Density Plot of Overall survival (OS)

Supplementary Table 1. Overall response rate for the whole cohort, and by response status
