# Supplementary material for: Clinico-molecular predictors of durable response to immune checkpoint inhibitors (ICI) in metastatic cervical cancer (mCC)
Source: Br J Cancer. 2026 May 19;135(4):581–7. doi: 10.1038/s41416-026-03438-6 (PMC13427743; doi:10.1038/s41416-026-03438-6)
Supplement: Supplementary file 1 — Supplementary Materials' legends [file 41416_2026_3438_MOESM1_ESM.docx]

**SUPPLEMENTARY MATERIALS, FIGURES AND LEGENDS**

Supplementary Table 1. Overall response rate for the whole cohort, and by response status.

NLTR = non long-term responders / LTR = long-term responders

CR = complete response, PR = partial response, SD = stable disease, PD = progression disease

Supplementary Figure 1. Kaplan–Meier curves for progression-free survival (PFS), defined according to the Response Evaluation Criteria in Solid Tumors, version 1.1. Tick marks indicate censored observations, and vertical lines indicate the times of landmark PFS analyses.

Supplementary Figure 2. Density Plot of Progression-Free Survival (PFS): The x-axis represents the time (in months/years) patients remain progression-free, while the y-axis represents the estimated probability density. The curve shows the distribution of PFS times among patients, indicating the relative likelihood of different survival durations. Peaks in the plot indicate the most common PFS times, and the spread of the curve indicates the variability in patient outcomes.

Supplementary Figure 3. Kaplan–Meier curves for overall survival (OS). Tick marks indicate censored observations, and vertical lines indicate the times of landmark OS analyses.

Supplementary Figure 4. Kaplan–Meier curves for overall survival (OS). Tick marks indicate censored observations, and vertical lines indicate the times of landmark PFS analyses.

LTR: Long term responders

NLTR: Non long term responders

Supplementary Figure 5. Density Plot of Overall survival (OS) The x-axis represents the time (in months/years) patients remain progression-free, while the y-axis represents the estimated probability density. The curve shows the distribution of PFS times among patients, indicating the relative likelihood of different survival durations. Peaks in the plot indicate the most common PFS times, and the spread of the curve indicates the variability in patient outcomes.
