# Supplementary figures and images for: Clinico-molecular predictors of durable response to immune checkpoint inhibitors (ICI) in metastatic cervical cancer (mCC)
Source: Br J Cancer. 2026 May 19;135(4):581–7. doi: 10.1038/s41416-026-03438-6 (PMC13427743; doi:10.1038/s41416-026-03438-6)

## Slide 1
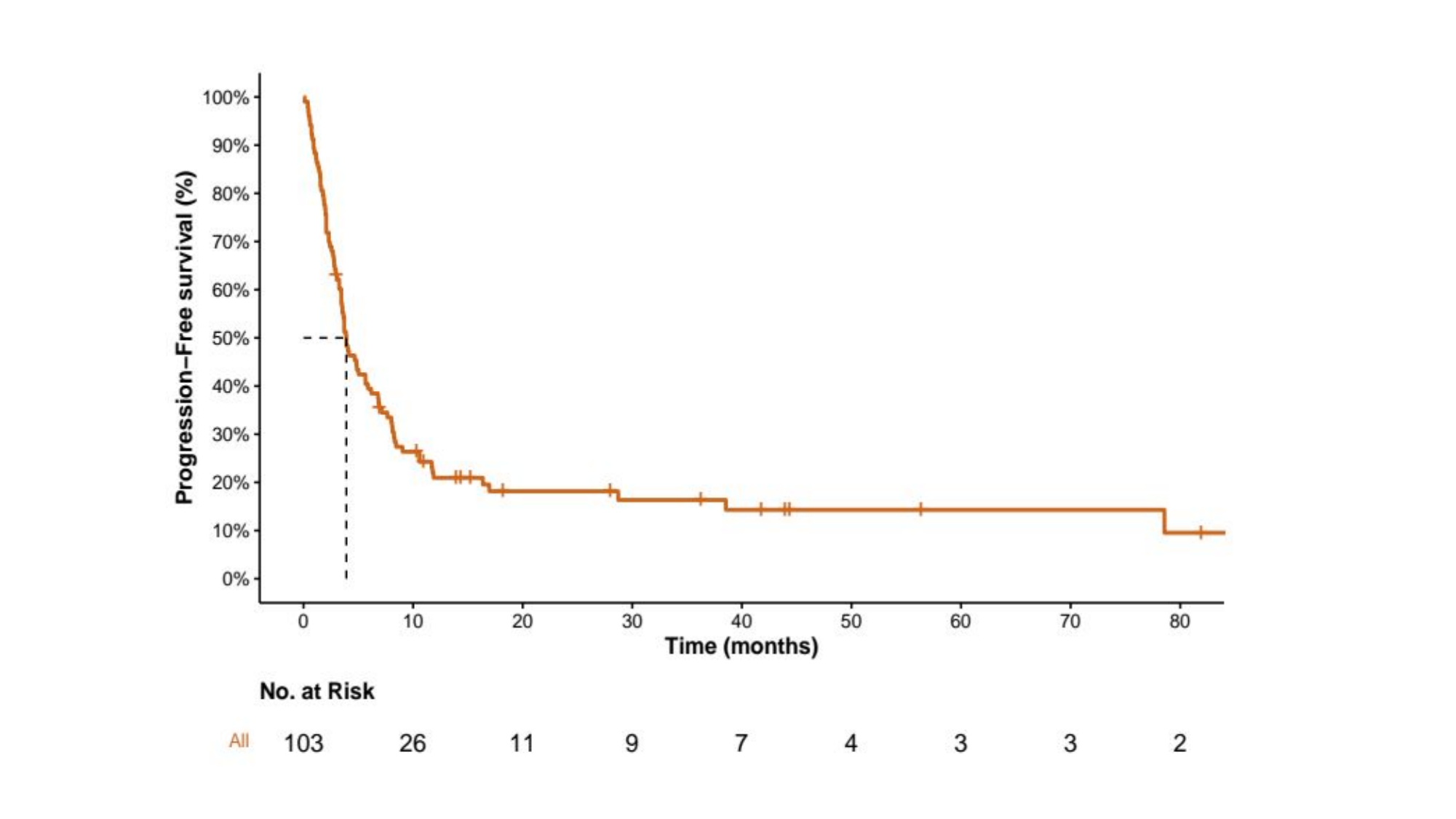

Supplement: Supplementary file 3 — Supplementary Figure 1. Kaplan–Meier curves for progression-free survival (PFS), defined according to the Response Evaluation Criteria in Solid Tumors, version 1.1. [file 41416_2026_3438_MOESM3_ESM.pptx]

## Slide 1
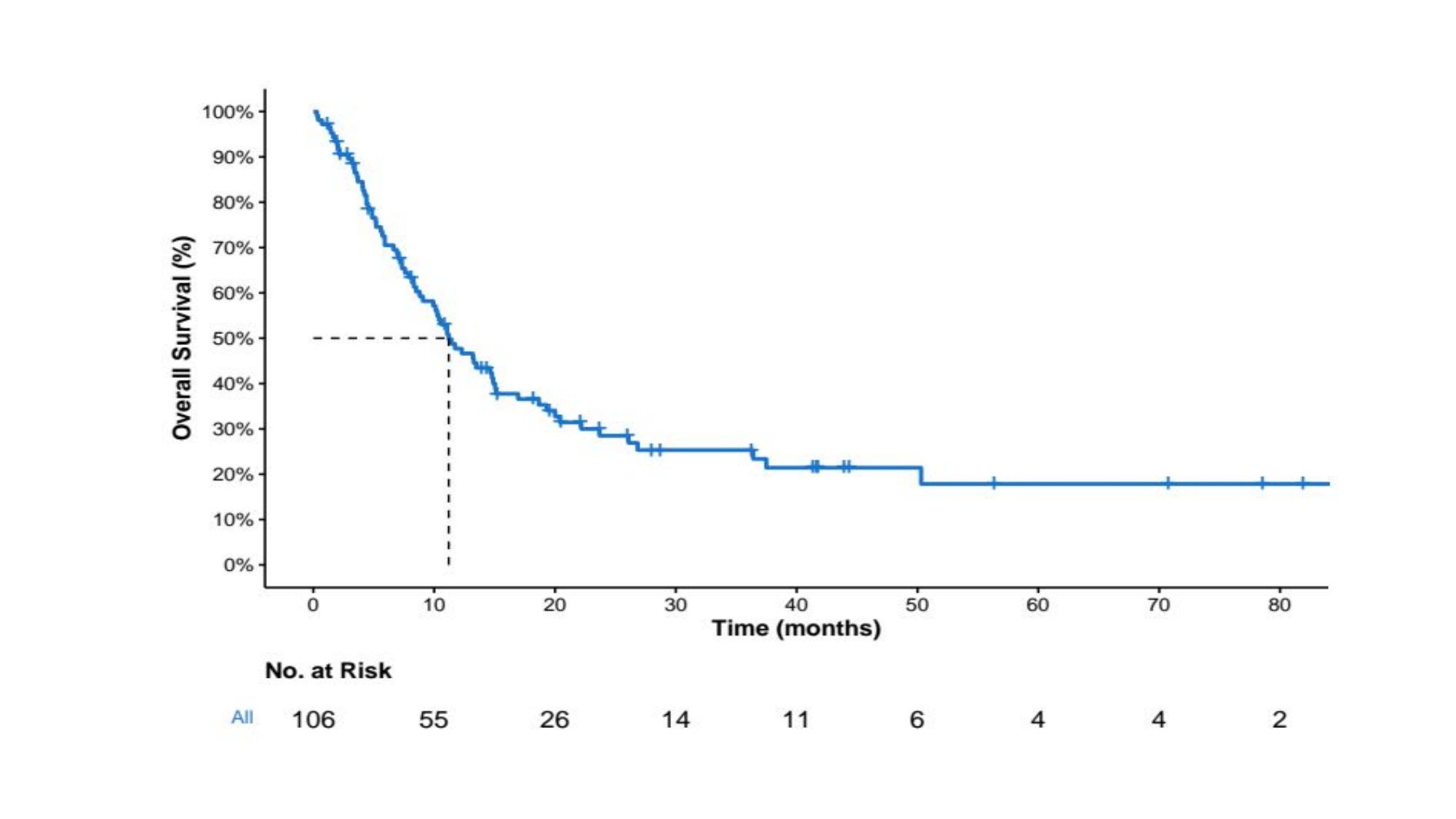

Supplement: Supplementary file 4 — Supplementary Figure 2. Density Plot of Progression-Free Survival (PFS) [file 41416_2026_3438_MOESM4_ESM.pptx]

## Slide 1
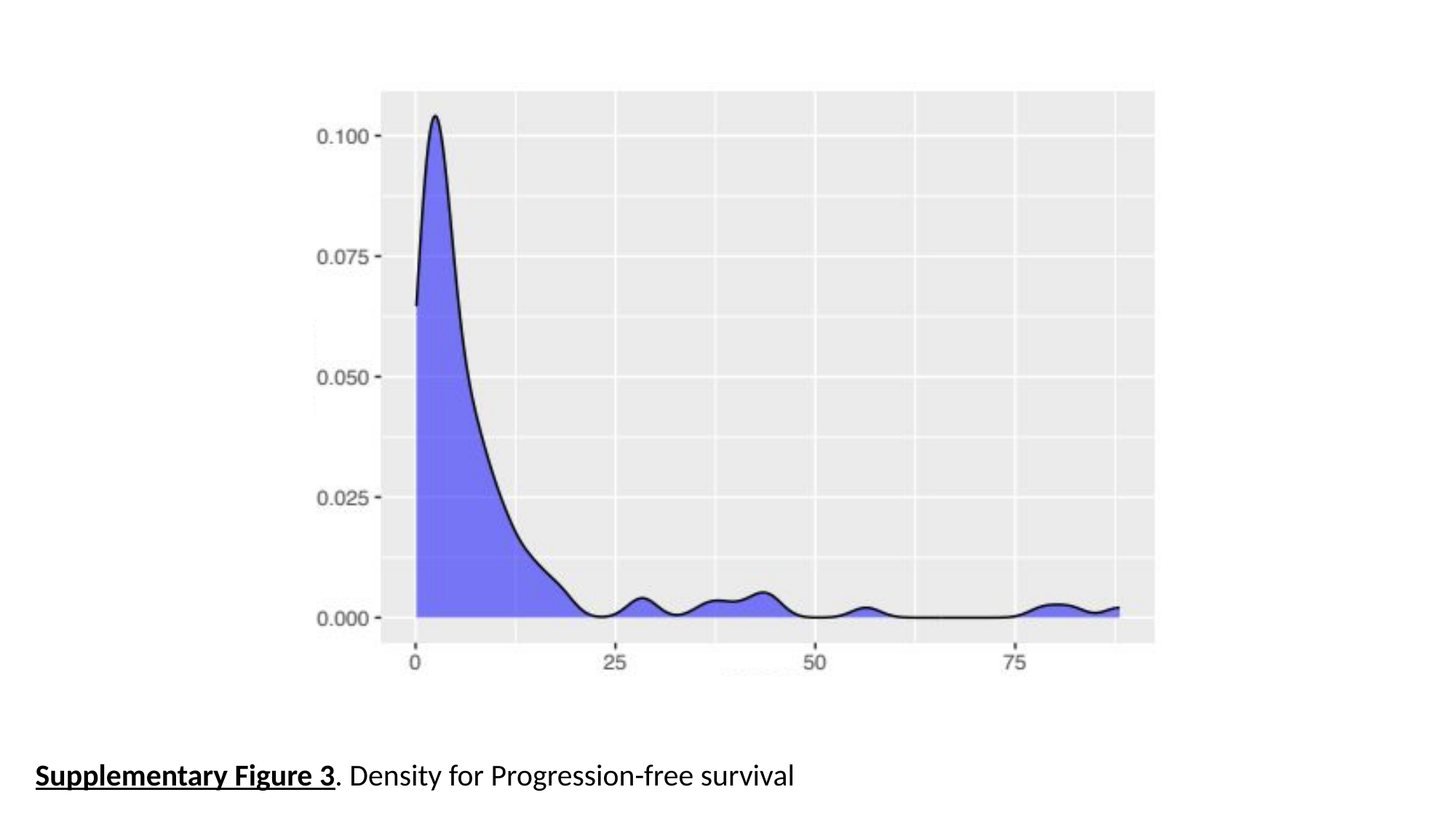

Supplementary Figure 3. Density for Progression-free survival

Supplement: Supplementary file 5 — Supplementary Figure 3. [file 41416_2026_3438_MOESM5_ESM.pptx]

## Slide 1
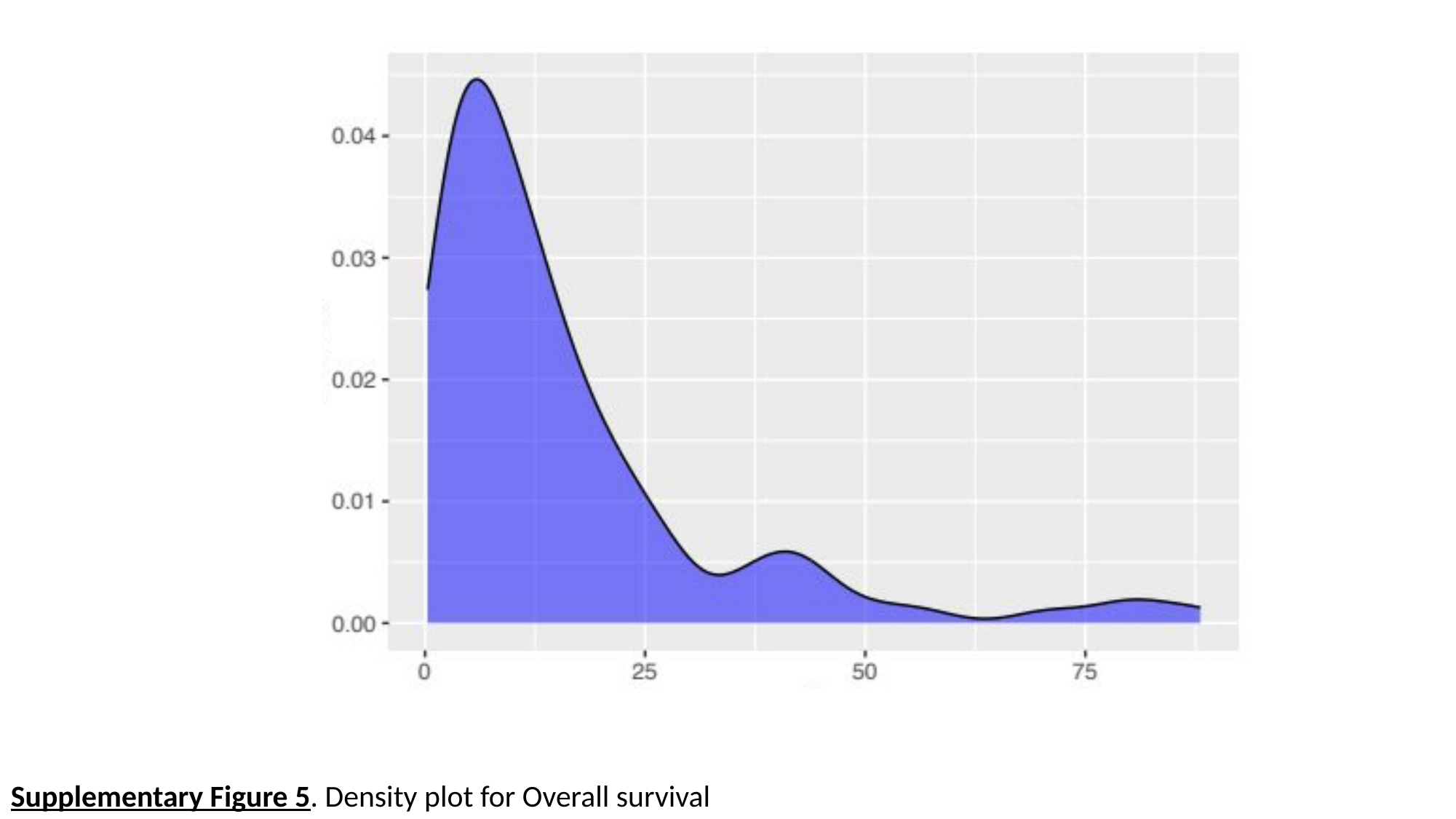

Supplementary Figure 5. Density plot for Overall survival

Supplement: Supplementary file 7 — Supplementary Figure 5. Density Plot of Overall survival (OS) [file 41416_2026_3438_MOESM7_ESM.pptx]
