# Supplementary material for: Clinico-molecular predictors of durable response to immune checkpoint inhibitors (ICI) in metastatic cervical cancer (mCC)
Source: Br J Cancer. 2026 May 19;135(4):581–7. doi: 10.1038/s41416-026-03438-6 (PMC13427743; doi:10.1038/s41416-026-03438-6)
Supplement: Supplementary file 6 — Supplementary Figure 4. Kaplan–Meier curves for overall survival (OS). [file 41416_2026_3438_MOESM6_ESM.pptx]

## Slide 1
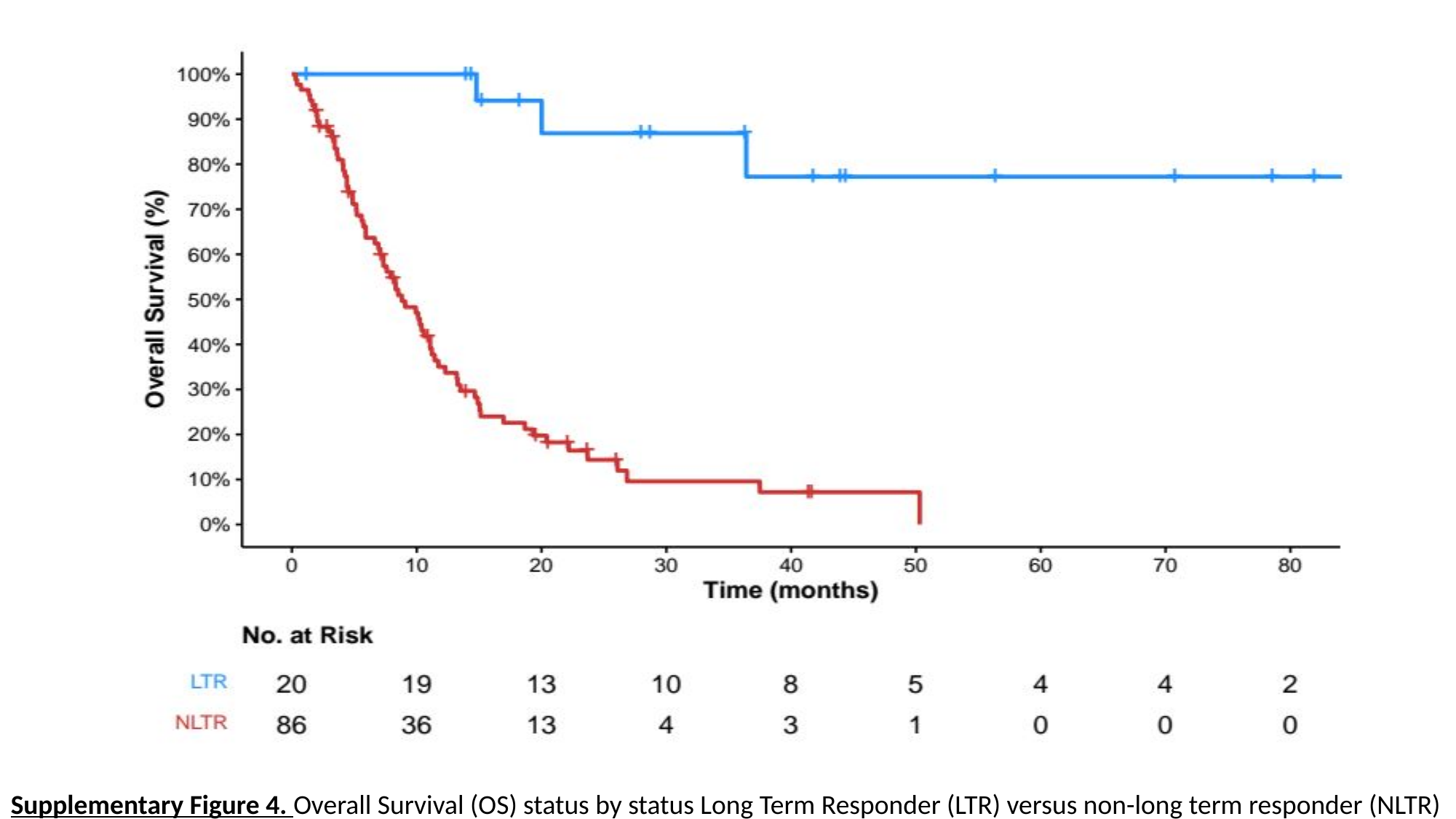

Supplementary Figure 4. Overall Survival (OS) status by status Long Term Responder (LTR) versus non-long term responder (NLTR)
